# Intergenerational impact of dietary protein restriction in dairy ewes on epigenetic marks in the perirenal fat of their suckling lambs

**DOI:** 10.1038/s41598-023-31546-3

**Published:** 2023-03-16

**Authors:** Pablo A. S. Fonseca, Aroa Suárez-Vega, Rocio Pelayo, Hector Marina, María Alonso-García, Beatriz Gutiérrez-Gil, Juan-José Arranz

**Affiliations:** Departamento de Producción Animal, Facultad de Veterinaria, Universidad de León, Campus de Vegazana S/N, 24071 León, Spain

**Keywords:** Animal breeding, Epigenomics

## Abstract

In sheep, nutrition during the prepubertal stage is essential for growth performance and mammary gland development. However, the potential effects of nutrient restriction in a prepuberal stage over the progeny still need to be better understood. Here, the intergenerational effect of maternal protein restriction at prepubertal age (2 months of age) on methylation patterns was evaluated in the perirenal fat of Assaf suckling lambs. In total, 17 lambs from ewes subjected to dietary protein restriction (NPR group, 44% less protein) and 17 lambs from control ewes (C group) were analyzed. These lambs were ranked based on their carcass proportion of perirenal and cavitary fat and classified into HighPCF and LowPCF groups. The perirenal tissue from 4 NPR-LowPCF, 4 NPR-HighPCF, 4 C-LowPCF, and 4 C-HighPCF lambs was subjected to whole-genome bisulfite sequencing and differentially methylated regions (DMRs) were identified. Among other relevant processes, these DMRs were mapped in genes responsible for regulating the transition of brown to white adipose tissue and nonshivering thermoregulation, which might be associated with better adaptation/survival of lambs in the perinatal stage. The current study provides important biological insights about the intergenerational effect on the methylation pattern of an NPR in replacement ewes.

## Introduction

Among sheep products, the meat of suckling lambs stands out as a high-quality product valuable in Mediterranean countries due to its tenderness, low-fat, pale pink color and moisture^1^. There is a protected geographical indication (PGI) known as ‘Lechazo de Castilla y León’ related to dairy sheep production in the northwest region of Spain [Commission Regulation (EC) No 2107/1999]. The animals which belong to this PGI are fed exclusively on sheep's milk and slaughtered before 35 days of age, at 9–12 kg of body weight. Consequently, in dairy flocks, suckling lambs are an important economic input. Suckling lambs have a rumen that is still not completely active; therefore, they can be considered functional monogastric organisms^2^. As a consequence, the composition of adipose depots in lambs is highly influenced by the diet they consume, for example, the milk and/or supplementary feedstuffs^3–9^. Therefore, the diet composition is a crucial factor for the proper development of the animal, resulting in a high-quality product that meets consumer demands. In general, feeding-related management decisions are responsible for the major cost in animal production systems, accounting for up to 75% of all variable costs in a herd, with protein corresponding to a high proportion of these costs^10–12^. In addition, the supply of protein for animal feed in Europe relies mainly on importing soybean from subtropical regions. This process might disturb the nutrient cycle due to the geographical separation of soybean cultivation and livestock production as the manure produced by the animals fed with the imported soybean will not be available to fertilize the land used for the plantation^13^. Additionally, the recent logistic crisis worldwide has drastically increased the prices of traditional sources of proteins, such as soybeans. Consequently, protein intake in flocks is a key point in the control of the sustainability of the production chain. Taken together with the market price volatility and availability of feed components, the feeding strategies are under constant pressure for changes and adaptations. A previous study by our group assessed whether nutritional protein restriction (NPR) performed at prepubertal age in Spanish Assaf ewe lambs would affect economically important traits^14^. Interestingly, no effect of the NPR was observed on the milk production potential and the somatic cell counts of the animals in response to an inflammatory challenge of the mammary gland, suggesting the possibility of reduction of protein intake in the diet of replacement ewe lambs without a negative impact on their production traits as adult ewes.

Fetal developmental programming is a consequence of maternal stimulus in a sensitive period of intrauterine development and results in permanent effects on the structure, physiology and metabolism of the fetus^15^. Maternal nutrition is a major environmental factor among the different stimuli capable of affecting fetal development. Different from suckling lambs, adult sheep have a fully functional rumen. Consequently, they can use all nitrogen (protein and nonprotein nitrogen) compounds as a feed source. In the rumen, the dietary rumen degradable nitrogen is converted mainly into ammonia, providing a proper environment for microbial fermentation and the flow of microbial amino acids. In Assaf rams, feed with a low protein concentration significantly affected scrotal circumference and body weight gain 11 weeks post-weaning^16^. In female sheep, protein restriction during the gestation period is associated with altered developmental patterns of the endocrine (response to chemical compounds such as noradrenaline and acetylcholine) and cardiovascular (vascular function) systems alongside changes in the growth pattern of the offspring^17,18^. Additionally, maternal nutrient restriction may result in small gestational age offspring with reduced muscle mass^19^ caused by reduced myofiber number and/or impaired hypertrophy^20^. At the gene expression level, the feed of dams during pregnancy with diets composed of fiber plus protein plus fat resulted in differences (> 200 differentially expressed genes in all the comparison groups) in the gene expression levels in the perirenal fat, subcutaneous fat and longissimus dorsi muscle of sheep fetuses^21^. In addition, the timing of nutrient restriction is well established as a disturbing factor of adipose tissue development^22^. In dairy sheep, nutrition during the prepubertal stage is an important factor for growth performance, mammary gland development and milk production in the later stages of development^23,24^. However, there is an evident lack of studies investigating the intergenerational impact of nutrient restriction during the prepuberal stage of dairy ewes.

Epigenetic modifications, which are molecular processes that do not change the DNA sequence and alter genome activity, can be responsible for these lifelong consequences on the progeny of parents subjected to feed restrictions^25^. Different molecular processes can be responsible for epigenetic alterations, such as RNA methylation, noncoding RNA expression, histone modification and DNA methylation^26–29^. The intergenerational transfer of maternal effects through DNA methylation is a relevant hypothesis that has the potential to help in the understanding of the biological processes associated with the effect of maternal stress on progeny. The effect on the DNA methylation profile of the offspring from a mother subjected to environmental stress, including nutritional restriction, has previously been described in different species^30–35^. More specifically, maternal nutrient restrictions are linked with persistent and intergenerational metabolic disturbances with effects on the health and survival of the offspring^36–38^. In suckling lambs, the perirenal and cavitary fat (PCF) represents a notable proportion of the total fat in the body, approximately 11% in suckling lambs from the Lechazo de Castilla y León protected geographical indication (PGI)^39^. In the first weeks of postnatal life, PCF is a very important component of the metabolism of lambs. The PCF transits from predominantly brown adipose tissue (BAT) in the first 4 days of life to predominantly white adipose tissue (WAT) at approximately 14 days of life^40^. However, in Assaf sheep, some studies from our research group confirmed the presence of brown adipocytes in the perirenal fat of suckling lambs slaughtered between 17 and 23 days of life^41^. The amount of BAT in the PCF is related to nonshivering thermogenesis, which is an important process for neonatal lamb survival^42,43^, and this transition from BAT to WAT reflects the necessity of the organism to move from thermoregulation in the first days of life to growth and homeostasis in the next stages of life. Interestingly, important components of the biological machinery responsible for nonshivering thermogenesis, such as *UCP1* (a biomarker of BAT), are associated with metabolic inefficiency^42^. Consequently, different levels of PCF in suckling lambs might be associated with different responses to NPR. It is important to highlight that, to our knowledge, no studies in dairy sheep have evaluated the intergenerational consequences of an NPR performed in prepubertal replacement ewes over the offspring genome methylation markers.

Based on the preceding information, the main objectives of the current study were: (1) to investigate differential methylation patterns across the genome between the progeny from ewes subjected to an NPR at prepubertal age and progeny from control ewes; (2) to evaluate the effects on the methylation pattern across the genome, considering the interaction between the protein restriction in the ewes and the perirenal fat content in the progeny; and (3) to estimate the isolated impact of divergent perirenal fat content on the methylation pattern across the genome of Assaf suckling lambs.

## Results

### Effects of carcass internal fat depot content and age across groups

The age and half-carcass percentage of PCF for all 34 male lambs born are available in Supplementary Table 1. The descriptive statistics for the same traits for the 16 samples assigned to the four groups defined based on the two considered factors (NPR-LowPCF, NPR-HighPCF, C-LowPCF, and C-HighPCF) are shown in Table 1. The Anderson–Darling normality test indicated that there was no relevant deviation from normality for age in days (*P* value = 0.640) and PCF (*P* value = 0.019). The exploratory analysis of age in days and PCF suggested differences between the groups from the nutritional challenge and fat content (Supplementary Fig. 1). Significant differences were observed for the percentage of perirenal fat between the HighPCF and LowPCF fat groups regardless of the nutritional challenge groups (*P* value = 6.85 × 10^–8^) and between the HighPCF and LowPCF fat groups within the nutritional challenge groups C (*P* value = 0.004) and NPR (*P* value = 0.0003). Therefore, the results suggest an effective grouping of animals in the high-fat and low-fat groups, allowing a potential detection of differential methylation between these groups. Only the comparison between the HighPCF and LowPCF fat groups, disregarding the nutritional challenge grouping, resulted in a significant difference for age in days (*P* value = 0.022). Therefore, the identification of differential methylation patterns between the NutChal and FatGroup groups was performed, including the age of the animal as a fixed effect.

**Table 1:** Summary statistics (mean ± standard deviation) for the half-carcass percentage of perirenal and cavitary fat (PCF) and age (in days) for the lambs from the nutritional challenge and fat groups.

	Nutritional protein restriction (NPR) and control (C) groups			
Fat group	Age (in days)		Percentage of perirenal and cavitary fat	
	NPR	C	NPR	C
HighPCF	28.00 ± 4.76	28.75 ± 6.34	3.25 ± 0.10	3.23 ± 0.47
LowPCF	22.25 ± 5.31	22.00 ± 4.08	1.67 ± 0.25	1.65 ± 0.16

*C* lambs which were born from control dams; *NPR* lambs which were born from nutritional protein restriction dams; *HighPCF* lambs which were assigned to the high PCF group; *LowPCF* lambs which were assigned to the low PCF group.

### Differentially methylated regions identified for the multifactorial design

The mapping statistics for the reads obtained in the WGBS are shown in Supplementary Table 2. An average mapping of 70.10 ± 2.55% was obtained, with values ranging from 65.56 to 75.58%. The mean percentage of methylated sites in the CG, CHG and CHH contexts (where H is A, C, or T) was 71.36 ± 0.5%, 1.40 ± 0.03%, and 1.47 ± 0.05% for the NPR group, respectively. Thus, the mean percentage of methylated sites for the CG, CHG and CHH contexts for the control group were 71.49 ± 0.36%, 1.42 ± 0.02%, and 1.49 ± 0.03%, respectively. Therefore, similar proportions of methylation in all three contexts were observed between the NPR and C groups.

**Figure 1:**
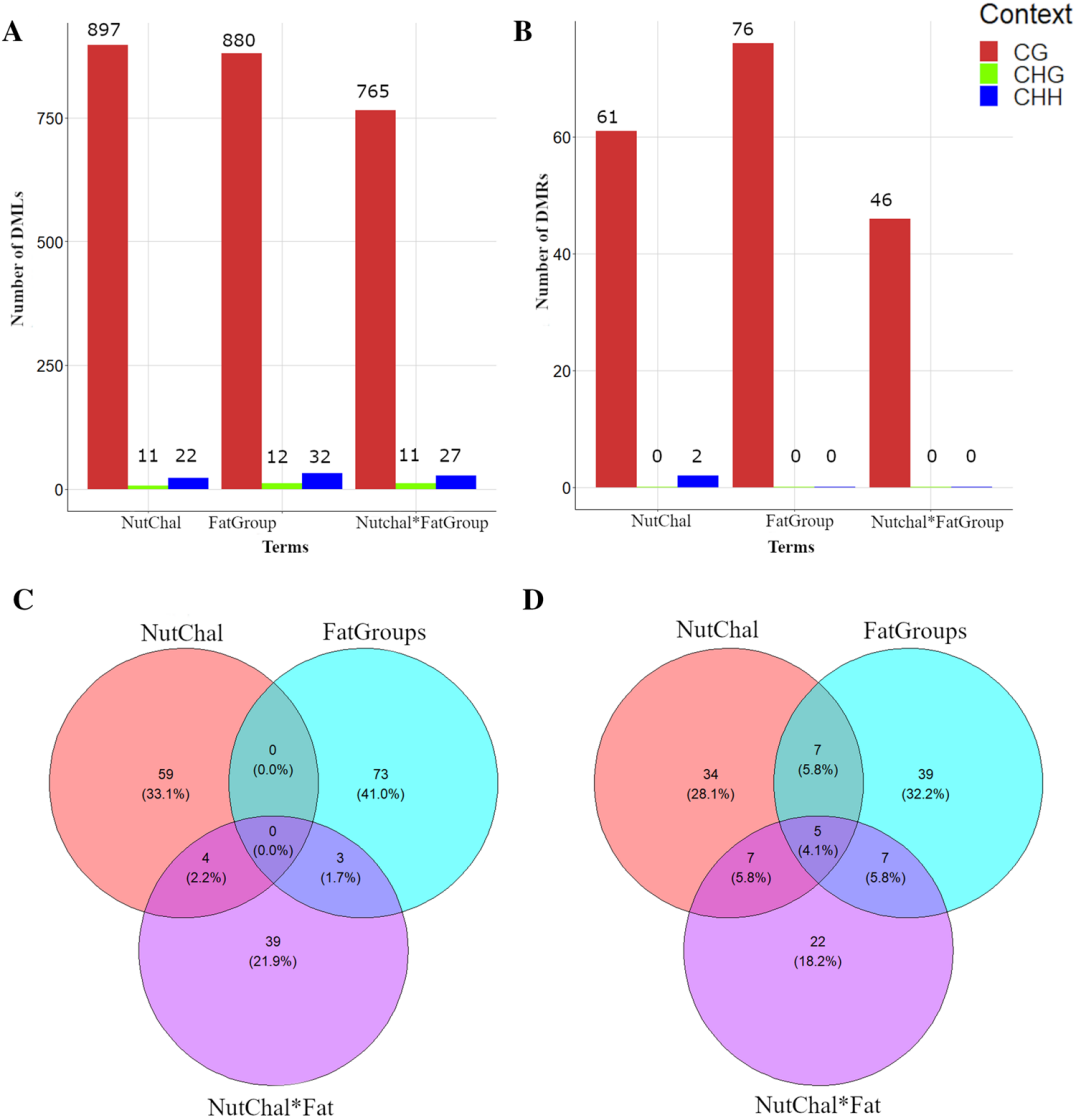
Bar plot showing the number of differentially methylated loci (**A**) and regions (**B**) identified in each nucleotide context (CG in red, CHG in green and CHH in blue) for the three terms evaluated in the multifactorial model (NuChal, FatGroup and NutChal * FatGroup). Venn diagram showing the number of differentially methylated regions (**C**) and genes harboring differentially methylated regions (**D**) among NuChal (ref), FatGroup (blue) and NutChal  *  FatGroup (purple).

The number of DMLs and DMRs for each term from the multifactorial model tested per methylation context are shown in Fig. 1. Except for the NutChal term, DMRs were identified only in the CG context, with 61 (in 53 genes), 76 (in 58 genes) and 46 (in 41 genes) DMRs identified for NutChal, FatGroups and NutChal  *  FatGroups, respectively (Supplementary Table 3). In addition, two DMRs were identified in the CHH context for the NutChal term. Interestingly, no DMRs were shared among all the terms (based on DMR start and end coordinates), while five genes harboring different DMRs were annotated and shared among the three terms (Fig. 1). The five shared genes were *ANXA2R* (annexin A2 receptor), *LOC121816055*, *TRNAG-GCC_6* (tRNA-Gly), *LOC114112700* (translation initiation factor IF-2-like), and *LOC121818805* (Table 2). In total, 24 unique DMRs were mapped in the regions harboring these five genes, with seven for *ANXA2R*, eight for LOC121816055, two for *TRNAG-GCC_*6, four for *LOC114112700*, and three for *LOC121818805*. The only shared DMR (NutChal  *  FatGroup and FatGroup) was mapped in the coordinates 1:112,885,986–112,886,049, harboring the *TRNAG-GCC_*6. Interestingly, only the DMRs identified in the *TRNAG-GCC_*6 gene were annotated in a promoter region among these 24 DMRs.

**Table 2:** Genes harboring differentially methylated regions and shared among all the three terms for the multifactorial model (Nutchal * FatGroup, NutChal, and FatGroup).

Coordinate	Gene	Length	nCG	Term	Context	Genomic context
16:31,783,099–31,783,205	*ANXA2R*	107	8	Nutchal * FatGroup	CG	Intergenic
16:31,790,384–31,790,436	*ANXA2R*	53	9	Nutchal * FatGroup	CG	Intergenic
16:31,727,079–31,727,262	*ANXA2R*	184	5	Nutchal * FatGroup	CG	Intergenic
16:31,731,294–31,731,346	*ANXA2R*	53	5	Nutchal * FatGroup	CG	Intergenic
16:31,787,826–31,787,932	*ANXA2R*	107	5	Nutchal * FatGroup	CG	Intergenic
2:250,153,162–250,153,236	*LOC114112700*	75	4	Nutchal * FatGroup	CG	Intergenic
1:120,946,293–120,946,367	*LOC121816055*	75	5	Nutchal * FatGroup	CG	Intergenic
2:249,907,223–249,907,543	*LOC121818805*	321	4	Nutchal * FatGroup	CG	Intergenic
1:112,885,986–112,886,049	*TRNAG-GCC_6*	64	5	Nutchal * FatGroup	CG	Promoter
16:31,807,558–31,807,655	*ANXA2R*	98	8	NutChal	CG	Intergenic
2:250,164,900–250,164,966	*LOC114112700*	67	7	NutChal	CG	Intergenic
2:250,145,061–250,145,120	*LOC114112700*	60	5	NutChal	CHH	Intergenic
1:120,986,211–120,987,063	*LOC121816055*	853	9	NutChal	CG	Intergenic
1:120,963,799–120,963,849	*LOC121816055*	51	5	NutChal	CG	Intergenic
1:120,985,694–120,985,753	*LOC121816055*	60	4	NutChal	CG	Intergenic
1:120,958,079–120,958,147	*LOC121816055*	69	4	NutChal	CG	Intergenic
2:249,927,235–249,927,294	*LOC121818805*	60	6	NutChal	CG	Promoter/Exon
1:112,885,986–112,886,049	*TRNAG-GCC_6*	64	5	NutChal	CG	Promoter
16:31,757,401–31,757,485	*ANXA2R*	85	5	FatGroup	CG	Intergenic
2:250,147,524–250,147,587	*LOC114112700*	64	4	FatGroup	CG	Intergenic
1:120,980,087–120,980,955	*LOC121816055*	869	11	FatGroup	CG	Intergenic
1:120,962,054–120,962,116	*LOC121816055*	63	9	FatGroup	CG	Intergenic
1:120,953,964–120,954,026	*LOC121816055*	63	8	FatGroup	CG	Intergenic
2:249,916,688–249,916,840	*LOC121818805*	153	13	FatGroup	CG	Intergenic
1:112,885,845–112,885,903	*TRNAG-GCC_6*	59	6	FatGroup	CG	Promoter

### Discriminant analysis between nutritional challenge and fat groups and functional interpretation of DMRs

**Figure 2:**
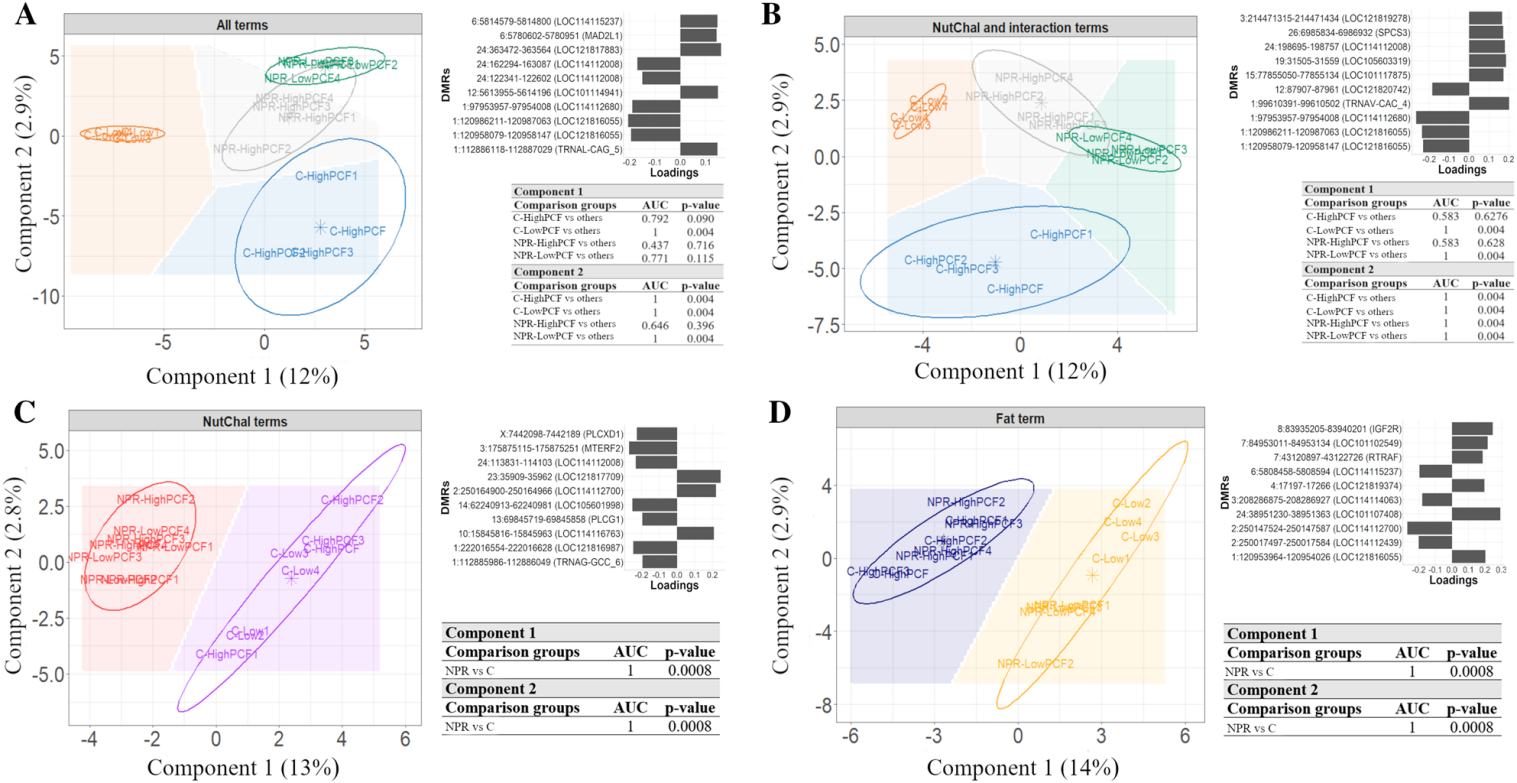
Results of partial least squares discriminant analysis (PLS-DA) and the top 10 loading vectors for the mean methylation levels within the identified differentially methylated regions (DMRs). (**A**) Discriminant analysis using the mean methylation level within the DMRs identified for the three terms of the multifactorial model (NutChal * FatGroup, NutChal, and FatGroup) to classify the four groups from the multifactorial model: lambs from control ewes with a low percentage of perirenal and cavitary fat (C-LowPCF in orange), lambs from control ewes with a high percentage of perirenal and cavitary fat (C-HighPCF in blue), lambs from nutritional challenge ewes with a low percentage of perirenal and cavitary fat (NPR-LowPCF in green), and lambs from nutritional challenge ewes with a high percentage of perirenal and cavitary fat (NPR-High in gray). (**B**) Discriminant analysis using the mean methylation level within the DMRs identified for only for the NutChal  *  FatGroup and NutChal terms to classify the four groups from the multifactorial model (C-LowPCF in orange, C-HighPCF in blue, NPR-LowPCF in green, and NPR-HighPCF in gray). (**C**) Discriminant analysis using the mean methylation level within the DMRs identified for the NutChal term terms to classify the NPR and C groups. (**D**) Discriminant analysis using the mean methylation level within the DMRs identified for the FatGroup term terms to classify the HighPCF and LowPCF perirenal and cavitary fat groups.

The PLS-DA using the methylation means simultaneously within the DMRs identified for all the terms from the multifactorial model resulted in poor discrimination of the samples from the nutritional challenge and high-fat groups (Fig. 2A). However, the exclusion of the DMRs exclusive to the FatGroup term resulted in perfect clustering (AUC = 1, *P* value = 0.004) for all the sample groups in the second principal component (Fig. 2B). In addition, the PLS-DA using the methylation levels within DMRs identified for the NutChal and Fat terms individually resulted in a perfect clustering of NPR versus C and HighPCF vs LowPCF groups, respectively (Fig. 2C,D). The mean methylation level for all DMRs used in the PLS-DA is available in Supplementary Table 4. Figure 2 shows the evaluation of the top 10 absolute loading values obtained in each discriminant analysis. All the loading vectors obtained in the PLS-DA for the DMRs evaluated are available in Supplementary Table 5.

The results of the QTL annotation for the DMRs identified in each term from the multifactorial model are shown in Supplementary Table 6. All enriched traits among the annotated QTLs within the coordinates of the DMRs identified for the NutChal  *  FatGroup term were associated with the QTL types of “Production” (body length, body height and chest girth) and “Meat and Carcass” (muscle depth at third lumbar). Two enriched traits were identified for the DMRs obtained for the NutChal term, milk fat yield (180 d) and total lambs born. Only one trait was enriched for the DMRs identified for the FatGroup, the muscle depth at the third lumbar, which belongs to the “Meat and Carcass” QTL type (Table 3). Interestingly, the same DMR (18:58,058,339–58,058,484) was mapped in the region associated with the muscle depth at the third lumbar for both NutChal  *  FatGroup and FatGroup terms.

**Table 3:** Enrichment results for the quantitative trait loci annotated within the genomic coordinates for the differentially methylated regions for all three terms of the multifactorial model (Nutchal * FatGroup, NutChal, and FatGroup).

Trait	Number annotated QTLs	Number of QTLs in the database	P-value	FDR	QTL type	Term
Body length	5	9	2.00 × 10^–08^	3.01 × 10^–07^	Production	NutChal * FatGroup
Body height	5	17	9.20 × 10^–07^	6.90 × 10^–06^	Production	NutChal * FatGroup
Chest girth	5	24	5.96 × 10^–06^	2.98 × 10^–05^	Production	NutChal * FatGroup
Muscle depth at 3^rd^ lumbar	2	15	0.013	0.032	Meat and Carcass	NutChal * FatGroup
Milk fat yield {180d}	4	276	0.007	0.014	Milk	NutChal
Total lambs born	3	173	0.013	0.018	Reproduction	NutChal
Muscle depth at 3^rd^ lumbar	2	15	0.004	0.037	Meat and Carcass	FatGroup

The GO terms annotated for the genes associated with the DMRs identified for each term of the multifactorial model are available in Supplementary Table 7. Enriched terms were obtained only for the genes associated with DMRs from NutChal * FatGroup (16 GO terms) and NutChal (one GO term) terms (Table 4). In general, the enriched GO terms identified for the NutChal  *  FatGroup interaction were associated with plasma membrane structure (*RASA3* and *MTSS2*), actin cytoskeleton (*SHROOM2*, *SYNE3* and *MTSS2*), lyase activity (*CA1* and *CA5A*), ligand-gated cation channel activity (*RASA3* and *SHROOM2*), and one-carbon metabolic process (*CA1* and *CA5A*). The only GO-enriched term identified for the DMRs identified for the FatGroup term was “phosphoric diester hydrolase activity” (supported by the *PLCG1* and *PLCXD1* genes).

**Table 4:** Enrichment results for the gene ontology annotated for the genes harboring differentially methylated regions for all three terms of the multifactorial model (Nutchal * FatGroup, NutChal, and FatGroup).

Description	Ontology	P-value	FDR	Genes	Term
Intrinsic component of the cytoplasmic side of the plasma membrane	CC	1.29 × 10^–05^	0.0006	*RASA3*, *MTSS2*	NutChal * FatGroup
Carbonate dehydratase activity	MF	4.17 × 10^–05^	0.0025	*CA1*, *CA5A*	NutChal * FatGroup
Cortical actin cytoskeleton	CC	0.0009	0.0202	*SHROOM2*, *MTSS2*	NutChal * FatGroup
Cortical cytoskeleton	CC	0.0016	0.0202	*SHROOM2*, *MTSS2*	NutChal * FatGroup
Cytoplasmic side of plasma membrane	CC	0.0039	0.0246	*RASA3*, *MTSS2*	NutChal * FatGroup
Cytoplasmic side of membrane	CC	0.0050	0.0246	*RASA3*, *MTSS2*	NutChal * FatGroup
Hydro-lyase activity	MF	0.0009	0.0272	*CA1*, *CA5A*	NutChal * FatGroup
Carbon–oxygen lyase activity	MF	0.0014	0.0272	*CA1*, *CA5A*	NutChal * FatGroup
Ligand-gated cation channel activity	MF	0.0029	0.0272	*RASA3*, *SHROOM2*	NutChal * FatGroup
Actin binding	MF	0.0032	0.0272	*SHROOM2*, *SYNE3*, *MTSS2*	NutChal * FatGroup
Ligand-gated ion channel activity	MF	0.0045	0.0272	*RASA3*, *SHROOM2*	NutChal * FatGroup
Ligand-gated channel activity	MF	0.0045	0.0272	*RASA3*, *SHROOM2*	NutChal * FatGroup
One-carbon metabolic process	BP	0.0003	0.0402	*CA1*, *CA5A*	NutChal * FatGroup
Lyase activity	MF	0.0080	0.0422	*CA1*, *CA5A*	NutChal * FatGroup
Actin filament binding	MF	0.0096	0.0445	*SHROOM2*, *SYNE3*	NutChal * FatGroup
Cell cortex	CC	0.0125	0.0461	*SHROOM2*, *MTSS2*	NutChal * FatGroup
Phosphoric diester hydrolase activity	MF	0.0023	0.0421	*PLCG1*, *PLCXD1*	NutChal

Despite the limited number of enriched terms, which could be expected due to the limited number of DMRs and associated genes identified, interesting functional profiles were obtained when the complete list of GO terms was analyzed. The network composed of genes and GO terms created for the genes annotated in each of the three terms suggested that several genes were connected through similar biological mechanisms.

**Figure 3:**
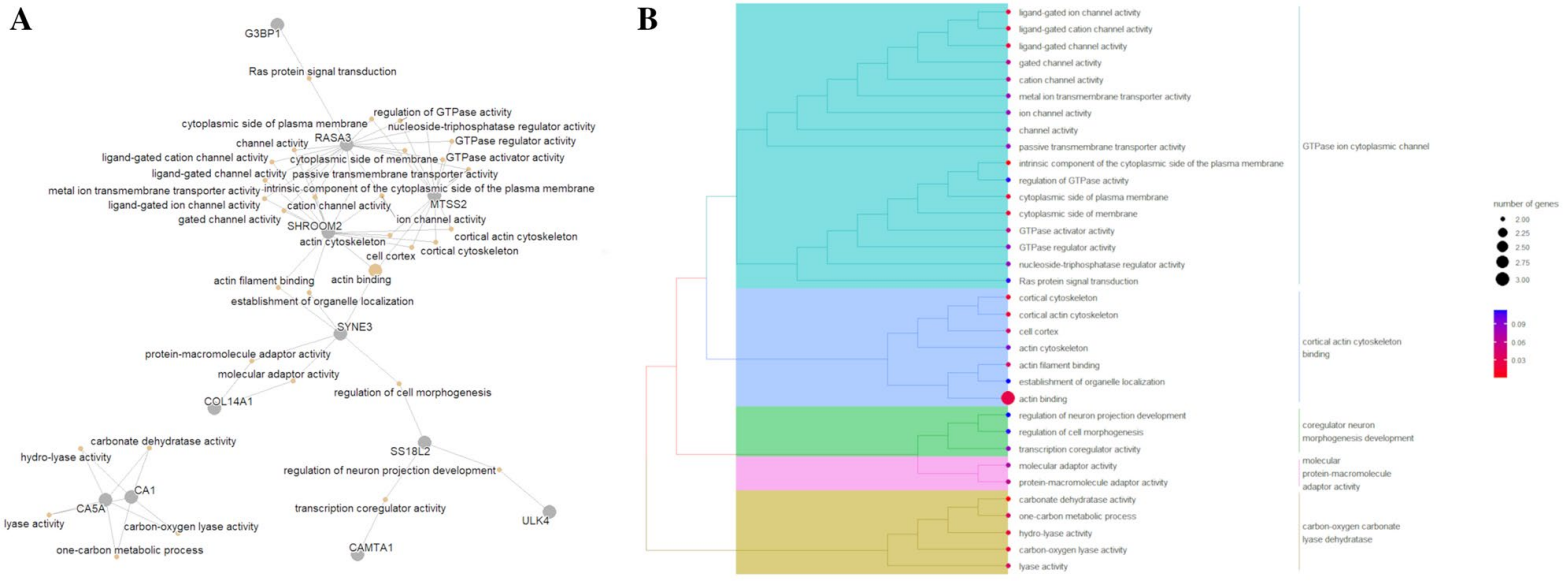
Functional analysis for the genes harboring differentially methylated regions for the interaction term (NutChal  *  FatGroup) of the multifactorial model. (**A**) Network composed of genes (gray circles) and gene ontology (GO) terms (yellow circles) showing the functional connection between the genes harboring DMRs identified for the interaction term (NutChal  *  FatGroup). (**B**) Functional grouping tree diagram for the annotated GO terms. Each color in the dendrogram represents a functional group obtained after estimating the Jaccard correlation coefficient. The area of the circles represents the number of genes assigned to each GO term, and the color of the circle indicates the P value estimated for each GO term.

The genes harboring DMRs identified for the interaction term of the multifactorial model (NutChal * FatGroup) were allocated into two principal clusters (Fig. 3). The largest cluster was composed of nine genes (*G3BP1, RASA3, MTSS2, SHROOM2*, *SYNE3*, *COL14A1, SS18L2*, *ULK4* and *CAMTA1*), while the second cluster was composed of *CA1* and *CA5A*. These clusters reflect the groups identified in the functional clustering of GO terms (Fig. 3B). The first cluster of genes was associated with the following functional groups of GO terms: “GTPase ion cytoplasmic channel”, “cortical actin cytoskeleton binding”, “coregulator neuron morphogenesis development”, and “molecular protein-macromolecule adaptor activity”. The functional grouping of GO terms associated with the second cluster indicated an association with the activity of “carbon–oxygen carbonate lyase dehydratase”.

Only the genes *PLCG1* and *PLCXD1* shared GO terms among the genes identified harboring the DMRs for the NutChal term in the multifactorial model. These genes shared the GO terms phosphoric ester hydrolase activity and phosphoric diester hydrolase activity (Supplementary Fig. 2). However, the functional grouping (including GO terms associated with only one gene) for the genes harboring the DMRs identified for the NutChal term suggested an association with interesting biological functions (Supplementary Fig. 2). Among these functional groups, it is interesting to highlight the “histone biosynthetic compensation chromosome” (*PCGF3* and *A4GALT*) and “glycolytic through fructose-6-phosphate glucose-6-phosphate” (*ADPGK*) terms.

**Figure 4:**
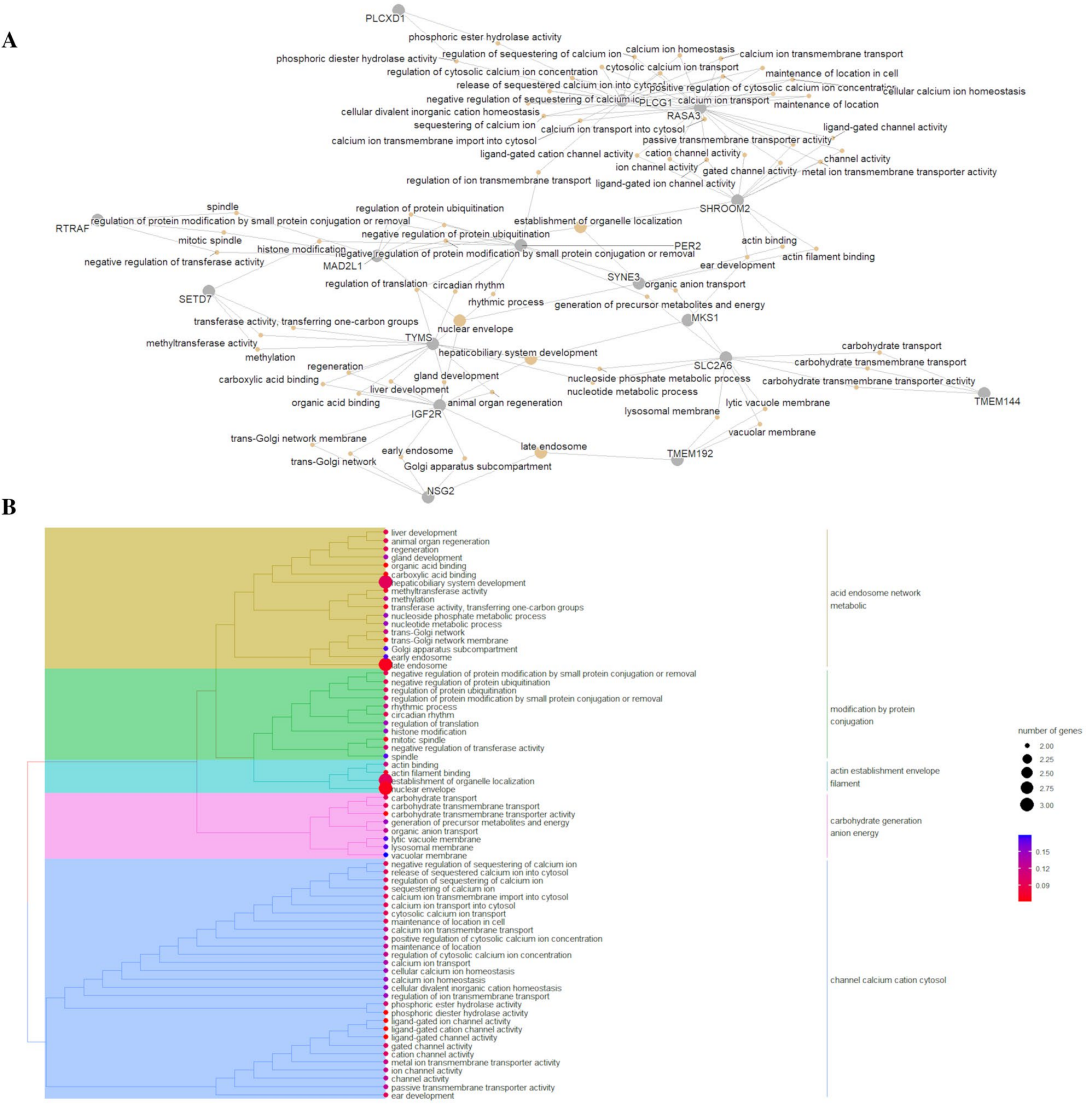
Functional analysis for the genes harboring differentially methylated regions for the FatGroup term of the multifactorial model. (**A**) Network composed of genes (gray circles) and gene ontology (GO) terms (yellow circles) showing the functional connection between the genes harboring DMRs identified for the FatGroup term. (**B**) Functional grouping tree diagram for the annotated GO terms. Each color in the dendrogram represents a functional group obtained after estimating the Jaccard correlation coefficient. The area of the circles represents the number of genes assigned to each GO term, and the color of the circle indicates the *P* value estimated for each GO term.

A single network was created for the genes harboring DMRs that were identified for the FatGroup (Fig. 4). This network was composed of 15 genes, from which 3 were identified in the network for the NutChal * FatGroup term (*RASA3*, *SHROOM2* and *SYNE3*), two were the genes interacting in the network for the NutChal term (*PLCXD1* and *PLCG1*), and 11 were exclusively identified for the FatGroup term (*MKS1*, *SLC2A6*, *TMEM144*, *TMEM192*, *PER2*, *NSG2*, *IGF2R*, *TYMS*, *MAD2L1*, *SETD7* and *RTRAF*). The functional grouping of the GO terms associated with the genes harboring DMRs that were identified for the FatGroup suggested activity over interesting biological processes (Fig. 4). The first functional group identified was “acid endosome network metabolic”, from which it is relevant to highlight liver development (*IGF2R* and *TYMS*), hepaticobiliary system development (*MKS1*, *IGF2R* and *TYMS*), animal organ regeneration (*IGF2R* and *TYMS*), gland development (*IGF2R* and *TYMS*) and methylation (*SETD7* and *TYMS*). The second functional group identified was “modification by protein conjugation”, which was composed of interesting GO terms, such as rhythmic process (*PER2* and *TYMS*), circadian rhythm (*PER2* and *TYMS*), histone modification (*SETD7* and *PER2*), regulation of translation (*PER2* and *TYMS*), negative regulation of protein ubiquitination (*MAD2L1* and *PER2*), and negative regulation of transferase activity (*MAD2L1* and *RTRAF*). The “actin establishment envelope filament” functional group is composed of GO terms such as actin filament binding (*SHROOM2* and *SYNE3*), the establishment of organelle localization (*MAD2L1*, *SHROOM2* and *SYNE3*) and nuclear envelope (MAD2L1, IGF2R and SYNE3). The “carbohydrate generation anion energy” functional group is composed of GO terms such as carbohydrate transmembrane transporter activity (*SLC2A6* and *TMEM144*), generation of precursor metabolites and energy (*SLC2A6* and *PER2*), organic anion transport (*SLC2A6* and *PER2*), and lytic vacuole membrane (*TMEM19* and *SLC2A6*). The last functional group identified was “channel calcium cation cytosol”, from which it is interesting to highlight the negative regulation of the sequestration of calcium ions (*PLCG1* and *RASA3*), phosphoric ester hydrolase activity (*PLCG1* and *PLCXD1*), and ligand-gated ion channel activity (*RASA3* and *SHROOM2*).

## Discussion

Increased attention to the intergenerational effects of maternal stress in the progeny has been observed for livestock species^44^. In cattle, the intergenerational effects of mastitis infection, heat stress and maternal metabolism have previously been evaluated^45–48^. In sheep, the multigenerational and intergenerational effects of maternal overnutrition were evaluated over leptin surge and metabolic syndrome, respectively^49–52^. In addition, the effect of parental diet on the progeny was described and confirmed in several species, such as humans, rodents and model organisms (*D. melanogaster* and *C. elegans*)^35,53^. However, to the best of our knowledge, there is no evaluation of the intergenerational effect of dietary protein restriction on methylation markers across the sheep genome. The intergenerational transfer of maternal effects through DNA methylation can help us to understand the biological processes involved in the response to protein restriction challenges. Therefore, in the current study, the effect of NPR in the prepuberal stage of Assaf ewes was evaluated regarding the impact over methylation marks across the genome of their suckling lambs. Here, DMRs were identified in functional candidate genes associated with relevant biological processes, which may help to better understand the effects in the offspring of ewes subjected to NPR in the prepuberal stage. Additionally, an interesting effect on the methylation marks regarding the interaction between the nutritional status of the ewes (NPR or C) and the level of PFC fat (high and low) in the lambs was observed.

Perirenal fat is the major fat deposit in suckling lambs, accounting for ~ 11% of total body fat (the rest of the fat is distributed across the subcutaneous, intermuscular and pelvic depots) and ~ 2% of total carcass weight in the Lechazo de Castilla y Leon PGI^39^. This tissue is very important for newborns due to its nonshivering thermogenesis ability and passes through an intensive transition from BAT to WAT^54,55^. This transition reflects the necessity of the organism to move from thermoregulation in the first days of life to growth and homeostasis in the later days of life^40,41^. Consequently, fine regulation of this tissue must be present to provide proper physiological conditions for survival and growth. Therefore, the study of methylation markers in the perirenal fat of suckling lambs is an interesting point to be scrutinized to better understand the biological processes involved with the intergenerational impact of dietary protein restrictions.

Despite being limited, the number of DMRs identified in the current study is in concordance with the number of DMRs identified in similar studies. For example, in an intergenerational study about the effect of methionine supplementation (a dietary methyl donor), 216 DMRs were identified in the sperm of F0 rams to produce the F1 generation^51^. However, it is important to highlight that here, a more stringent threshold (FDR < 0.05) was applied when compared to the threshold established in the abovementioned study (*P* value < 0.1). Consequently, the threshold reinforces the detection power of the experimental design and statistical model applied here.

### Genes shared among the different terms analyzed

Despite the absence of common DMRs among the three evaluated terms, five common genes harboring different DMRs were identified. Two of these genes were uncharacterized loci (*LOC121818805* and *LOC121816055*) encoding lncRNAs, and one was a LOC predicted as a translation initiation factor IF-2-like (*LOC114112700*). There is no functional information associated with any of these tree genes. *ANXA2R* encodes a receptor of annexin-2, a calcium-dependent protein that plays several roles in hematopoiesis, osteoclastic activation and osteoblast mineralization^56–58^. The fifth gene common among all three terms was *TRNAG-GCC_6*, which encodes a tRNA for glycine. The DMRs identified for this gene were mapped in the promoter region for all three terms. Glycine is a nonessential amino acid that may directly impact fetal development if major fetal requirements are not met^59^. Interestingly, during pregnancy in rats, supplementation with glycine rectifies the vascular dysfunction induced by protein restriction^60^. Additionally, an intergenerational effect of increased systolic blood pressure caused by a maternal low-protein diet was reversible through supplementation with glycine in rats^61^. In addition, in rats, protein restriction in dietary isocaloric diets resulted in higher levels of glycine in the liver, skeletal muscle, and kidney^62^. Consequently, these results indicate an important role of glycine in response to the effects caused by dietary protein maternal restriction, suggesting a potential functional role of the identified DMRs.

### Genes harboring DMRs identified for the Nutchal  *  FatGroup interaction term

The DMRs identified for the interaction term (Nutchal  *  FatGroup) were mapped, more than randomly expected (enriched), in regions previously associated with body composition traits in sheep (body length, body height, chest girth, and muscle depth at the third lumbar). Two DMRs were associated with these traits, which were mapped in the following genomic coordinates: 11:50,031,219–50,031,384 18:58,058,339–58,058,484. The first DMR was mapped in intronic or exonic regions (depending on the transcript isoform) in a CG context of the genes *LOC121820642* and *SYNE3*, respectively. *LOC121820642* is a lncRNA with no available functional information. *SYNE3*, also called Nesprin-3, is a member of a family of nuclear transmembrane proteins that binds to the cytoskeletal linker protein plectin, helping to regulate endothelial cell morphology and perinuclear cytoskeletal architecture^63,64^. Other members of the nesprin family (nesprin-1 and nesprin-2) are associated with muscle development and myogenesis^65,66^. The major functional group observed in the analysis performed using the Jaccard coefficient similarity matrix for the genes harboring DMRs identified for the NuChal * FatGroup term was “GTPase ion cytoplasmic channel”. The genes *RASA3*, *G3BP1*, *MTSS2* and *SHROOM2* were associated with this functional group. Among these genes, *RASA3* encodes Ras p21 protein activator 3, a stimulator of GTPase activity of Ras p21 and was previously identified as a GTPase-activating protein modulating Ras activity during normal brain development^67^. Interestingly, the Ras p21 protein is involved in the progression of fetal brown adipocytes through the S phase of the cell cycle in rats^68^. Additionally, Ras signaling regulates the differentiation of brown adipocytes and *UCP1* expression^69^. A key function of *UCP1* in the thermogenesis of brown adipocyte tissue is highlighted by the fast abundance decrease of its mRNA levels after birth, following the BAT decrease, acting in a protective manner to increase the survival rates in the neonatal period^43,70,71^. Consequently, *UCP1* is considered a classical biomarker for BAT. Indeed, the percentage of multilocular adipocytes in Assaf sheep is followed by the expression of UCP1, as recently demonstrated by our research group^41^. It is important to highlight that in the current study, for the interaction term, the DMR mapped on *RASA3* was located in the promoter region. Among the other genes harboring DMRs identified exclusively for the interaction term (Nutchal  *  FatGroup), *COL14A1* is involved in the development of muscle and preadipocytes^72^. The expression and polymorphisms (near or within the genomic coordinates) of *COL14A1* were associated with fat content in pigs and cattle^73–76^. In addition, *COL14A1* is mapped in regions of the cattle genome previously identified as signature selections for growth efficiency^77,78^. The second cluster of genes, including *CA1* and *CA5A*, was associated with one-carbon metabolism. Interestingly, methylation in DNA occurs as an output of one-carbon metabolism through the metabolic pathway responsible for the utilization of dietary methyl donors, usually obtained from folate transfer of methyl groups obtained from methionine^79^.

### Genes harboring DMRs identified for the Nutchal term

The genes harboring DMRs identified exclusively for the NutChal term from the multifactorial model and sharing GO terms, *PLCG1* and *PLCXD1*, were associated with phosphoric ester hydrolase activity and phosphoric diester hydrolase activity. Interestingly, the same GO terms were identified as enriched in the analysis of differentially expressed genes in the subcutaneous fat from the progeny of ewes fed with dried corn distillers grains (fiber plus protein plus fat) when compared with the progeny of ewes fed with alfalfa haylage (fiber) or corn (starch)^21^.

The analysis of the DMRs identified exclusively for the NutChal term was supposed to pinpoint candidate genes associated with biological functions not related to the differential fat content present within the NutChal and control groups. The major functional group identified for this term was the “histone biosynthetic compensation chromosome”. This functional group was related to biological processes involved in the regulation of gene expression by genome imprinting, histone ubiquitination, and neuroepithelial and odontoblast differentiation. In total, 53 genes harboring DMRs identified for the Nuchal term were annotated, of which 33 were uncharacterized loci. Consequently, the functional interpretation of these genes is difficult to apply. Among the other 20 genes, five codifying tRNAs were annotated (*TRNAV-CAC_4*, *TRNAG-GCC_6*, *TRNAS-GGA_58*, *TRNAS-GGA_161*, and *TRNAW-CCA_86*), which suggests an impact of protein restriction on the general availability and/or use of amino acids in the progeny. Maternal protein restriction was previously associated with a low level of circulating amino acids during intrauterine growth^80^. In addition, low amino acid levels were observed in newborns affected by intrauterine growth restriction caused by maternal protein restriction^81^. Consequently, this reinforces the potential role of these methylation marks as a response to protein restriction and subsequent amino acid availability.

### Genes harboring DMRs identified for the FatGroup term

In total, 11 genes were annotated exclusively for the DMRs identified for the FatGroup term (*MKS1*, *SLC2A6*, *TMEM144*, *TMEM192*, *PER2*, *NSG2*, *IGF2R*, *TYMS*, *MAD2L1*, *SETD7* and *RTRAF*). The analysis of biological functions to which these genes are enrolled might be useful to understand epigenetic differences observed between the sheep with divergent fat content, excluding the effect of the nutritional challenge or control groups.

A gene identified exclusively for the FatGroup term associated with adipocytes was *IGF2R*, which has a DMR mapped in the promoter, exonic or intronic regions (depending on the transcript). In cell culture, the knockdown of *IGF2R* affects the survival of brown adipocyte precursor cells negatively and reduces brown adipogenesis^82^. Interestingly, *IGF2R* is mapped in a cluster of genes with differential imprinting patterns between the maternal and paternal DNA in the progeny. The paternally expressed transcripts act as enhancers of prenatal growth, and the maternally expressed transcripts act as inhibitors of prenatal growth in mice^83^. Additionally, in pigs, *IGF2R* was identified as differentially expressed between pig breeds (Songliao black and Landrace) with extreme levels of backfat (high and low), with a higher expression in the low backfat breed (Landrace)^84^. In addition, *IGF2R* mutations are associated with perinatal lethality and overgrowth^85,86^. Consequently, these results reinforce the potential effect of the DMRs mapped within *IGF2R* on differential fat content and survival rate in Assaf suckling lambs.

Another functionally relevant gene for differential fat deposition was the *PER2* (period 2) gene, which has a DMR mapped in one of its introns. The *PER2* gene plays an important role in the regulation of the circadian clock, generating the circadian rhythm in the central nervous system and peripheral organs^87^. Interestingly, circadian rhythms are associated with the control of lipid metabolism^88^. More specifically, *PER2* is responsible for directly regulating *PPARγ*, a nuclear receptor that plays crucial roles in adipogenesis, the inflammatory response and insulin sensitivity^89–91^. This regulation occurs through the repression of *PPARγ* by blocking target promoters and transcription factors^92^. Additionally, mice deficient for *PER2* were observed to show a drastic reduction in total triacylglycerol and nonesterified fatty acids^92^. Interestingly, in sheep, the suppression of melatonin by exposure to constant light resulted in increased basal lipolysis with overexpression of adipogenic/thermogenic and circadian clock genes (including *PER2* and *PPARγ*)^93^. In addition, in the same experiment, the weight of BAT was half of the weight observed in the newborns of ewes supplemented with melatonin^93^. These results corroborate the physiological importance of the circadian clock to the regulation of BAT^94^. In cattle, the silencing of *PER2* was associated with suppressing lipid synthesis in the mammary gland through the regulation of *SREBF1* and *PPARγ*^95^. The results obtained here first suggest a potential impact of methylation markers on *PER2* over differential fat deposition in sheep.

The intergenerational effects of nutrient protein restriction during the prepubertal stage of replacement ewes, especially protein restriction, on methylation markers across the genome of livestock species are poorly evaluated. In the current study, the effects of protein restriction performed in replacement ewe lambs and the interaction with the amount of fat in the perirenal and cavitary deposits of their progeny were evaluated using WGBS from perirenal tissue. The results obtained here suggest that differential methylation is caused in suckling lambs by maternal protein restriction at prepubertal age. In addition, a multifactorial model was employed to identify DMRs for the interaction between the nutritional challenge and divergent fat level deposition groups and individually for the nutritional challenge and divergent fat deposition groups. The PLS-DA analysis confirmed that these DMRs could perfectly classify those groups. The functional analyses of the genes harboring these DMRs suggested their involvement in relevant biological processes. The DMRs identified individually for nutritional challenge groups were mapped in several tRNAs from different amino acids, suggesting a relationship between a dietary protein restriction of replacement ewe lambs and the availability of amino acids in their progeny. In addition, the genes harboring DMRs identified for the interaction between the nutritional challenge performed here and the divergent fat deposition groups were associated with the development and differentiation of adipocytes, especially those that characterize BAT. Similarly, the DMRs identified individually for the divergent fat deposition groups were also associated with the regulation of adipocytes and BAT. However, this regulation seems to occur by a different mechanism, where the regulation of the circadian rhythm is involved. In light of the above, the current study provides important biological insights into the effect of protein restriction in replacement ewes on the resulting methylation pattern in their future progeny. These results, consequently, may help pinpoint potential candidate genes and biological processes involved with different phenotypes regarding fat deposition and feed utilization.

## Material and methods

### Ethical approval

All procedures involving animals in this study were performed in accordance to Spanish regulations regarding the protection of animals used for experimental and other scientific proposes (Royal Decree 53/2013), under the supervision of the Ethical and Animal Welfare Committee of the University of León to after the approval of the competent body, Junta de Castilla y León. The nutritional challenge described in this work (ewes) was approved by the Ethics Committee of the Instituto de Ganadería de Montaña (IGM, CSIC-ULE) in León (Spain) (Reference 100102/2018-1).

Regarding their suckling lambs, the management in a commercial farm, the transport and sacrifice were performed following Spanish and EU legislation [Spanish Laws 32/2007, 6/2013 and RD 37/2014; Council Regulation (EC) 199/2009]. According to the Research Ethics Committee of the University of León, formal ethical approval was not necessary for this case. In addition, the experimental design and the analysis performed in the current study are in accordance with ARRIVE guidelines^96^.

### Nutritional protein restriction experiment for ewes and selection of lambs with divergent internal fat deposition levels

Initially, 40 Assaf female lambs (2 months of age) were acquired from one flock in the northwest region of Castilla y León (Spain) and transported to the facilities of the Instituto de Ganadería de Montaña (IGM, CSIC-ULE) in León. All the animals were fed a standard diet for replacement ewe lambs providing 16% crude protein until three months old, and subsequently, they were divided into two groups. These two groups were composed of 20 nutritionally challenged (NPR) and 20 control (C) animals to evaluate the impact of a feed restriction challenge due to a trade market problem and a shortage of concentrate inputs. Over 64 days, the C ewes received the standard diet mentioned above, while the NPR ewes received the same diet without soybean meal (44% reduction in protein intake). The complete description of the standard and NPR diets can be found at Supplementary Table 8^97^. After that period, the two groups received the same diet (standard diet) according to their growing needs and physiological status. At ten months of age, the ewes were artificially inseminated and subjected to standard periodic veterinary treatments (e.g., vaccines and anthelmintic treatments) and routine monitoring of their health status.

This study initially considered a total of 34 male lambs born in the same lambing season (January and February 2020) from the ewes included in the NPR experiment. These lambs were kept with the dams during the first four to eight hours of life to suck the first colostrum. Subsequently, the lambs were fed with milk replacer powder ad libitum using a milk replacer machine until slaughter. The lambs were slaughtered at a local slaughterhouse when the market weight was reached, with an average age of 24.85 ± 5.25 days (range of 16–37 days) and an average carcass weight of 2.87 ± 0.3 kg (range of 2.36–3.80 kg). At slaughter, perirenal adipose tissue was collected from each of the sacrificed lambs. The tissue samples were frozen at − 20 °C until DNA extraction.

With the aim of considering in our study the potential interaction of the effects of the nutritional challenge of the dams and the internal fat depot levels of the lambs, the percentage of PCF was measured in the half carcasses of all 34 slaughtered lambs. The age and PCF values for all 34 male lambs born are available in Supplementary Table 1. Based on the ranked distribution of the PCF values within the NPR/C groups, we selected the four animals with the highest (H) and lowest (L) PCF values born from the NPR and C dams. Consequently, a total of 16 Assaf suckling lambs were selected for further analyses related to the identification of methylation marks between the NPR and C lambs (4 NPR-LowPCF, 4 NPR-HighPCF, 4 C-LowPCF, and 4 C-HighPCF). Descriptive statistical analysis for the PCF trait in relation to the NPR and C groups was performed for these 16 selected animals using the R 4.2.0 statistical environment^98^. Additionally, as the animals were slaughtered at different ages, we evaluated the effect of age on the PCF trait. Initially, the Anderson–Darling normality test was applied to identify potential normality deviations. After this step, a test of variance equality was applied, and a t*-*test was chosen to test the differences in age and percentage of perirenal and cavitary fat across the groups. Significant differences between groups were defined using a threshold of *P* value < 0.05.

### DNA extraction and whole-genome bisulfite sequencing analysis

The perirenal fat from the 16 selected animals was used for DNA extraction using the QuickGene DNA Tissue Kit (Autogen, MA, USA), which is based on protein removal by protease K following the manufacturer’s instructions. The whole-genome bisulfite sequencing (WGBS) protocol applied to these samples was previously described by reference^99^. Briefly, the samples were used for paired-end (150 bp) library construction on Novogene in Cambridge (UK), and libraries were sequenced on an Illumina NovaSeq 6000, with a minimum coverage depth of 20X for each sample. The raw datasets derived from the sequencing are available at the European Nucleotide Archive (ENA) repository with accession number PRJEB56595.

### Methylation calling and identification of differentially methylated regions

The WGBS raw reads generated for the 16 samples of perirenal fat tissue under analysis were subjected to quality control using FastQC^100^.(Andrews, 2015),Subsequently, the reads were trimmed based on quality scores (Phred < 20), the adapters were removed, and short reads (< 20 bp) were filtered using the default options of Trim Galore software (version 0.6.5)^101^.

The trimmed reads were aligned to the reference genome (Oar_Ram_v2.0), indexed by BowTie2^102^, using Bsseeker2 software by the Python script bs_seeker2-align.py with the default options. The alignment output files were sorted by position using Samtools software (version 1.15.1)^103^, and the duplicated reads were removed using Picard software (version 2.25) (https://broadinstitute.github.io/picard/). Finally, the methylation call procedure was performed using the Python script bs_seeker2-call_methylation.py from Bsseeker2 using the default options.

The differentially methylated loci (DMLs) and DMRs were identified using the R package DSS^104^. Only methylated sites with ten or more reads mapped within the region were included in the analysis. First, a simple average algorithm for smoothing was used to estimate the mean methylation levels. A multifactorial design was applied to identify the DMLs using a model that included the NPR and control groups, the age of the animals (in days) and an interaction between the nutritional challenge and fat groups (NutChal * FatGroups) as terms. Subsequently, DMRs were detected based on regions harboring statistically significant methylated sites based on the following criteria: False-Discovery Rate (FDR) adjusted *P* value < 0.05 for the methylated site, minimum length (50 bp), minimum number of methylated sites (50 bp), and percentage of methylated sites being significant in the region (0.5). Additionally, the DMRs mapped in regions less than 50 bp from each other were merged into a single DMR.

### Partial least squares discriminant analysis between nutritional challenge and fat groups

The R package mixOmics^105^ was used to perform a partial least squares discriminant analysis (PLS-DA) to evaluate the potential of the identified DMRs to discriminate between the groups from the Nutchal and FatGroups effects. First, for each term from the multifactorial model used to identify the DMRs (NutChal, FatGroups, and NutChal  *  FatGroups), the average methylation level for the DMLs within each DMR was calculated. The discriminant potential was evaluated through the area under the curve (AUC) values and graphically plotting the two first principal components with centroids defining the groups.

### Annotation of gene ontology terms, metabolic pathways and QTLs associated with the differentially methylated regions

The genes harboring the identified DMR were annotated with the R package genomation^106^ using the ovine reference genome Oar_Ram_v2.0. The R packages ClusterProfiler^107^ and enrichplot were used for Gene Ontology (GO) term annotation, graphic representation and functional grouping. GO terms and KEGG pathways^108,109^ were considered enriched when the FDR was < 0.05, and a minimum of two genes were assigned to the respective term. The function pairwise_termsim() from enrichplot was used to calculate the Jaccard correlation coefficient among GO terms. After this step, GO terms shared between at least two genes were functionally grouped using the Jaccard correlation matrix to identify classes of terms that were closely related. For the functional grouping, the GO terms were included to estimate the Jaccard correlation matrix, disregarding the enrichment status. In addition, the GO terms shared between at least two genes were used to create networks linking different genes through the GO terms shared using the cnetplot() function.

The GALLO R package^110^ was used to annotate the colocation of the genes harboring DMRs with quantitative trait loci (QTL) using the SheepQTLdb from Animal QTLdb^111^ and considering a 250 kb interval downstream and upstream. The gff file from SheepQTLdb (Oar_Ram_v2.0) was edited to remove QTLs with lengths higher than 1 Mb. This approach was chosen to avoid the annotation of QTLs, which cover large extensions of the chromosomes, usually identified by linkage methods and using low-density microsatellite marker maps. In addition, a QTL enrichment analysis was performed using the qtl_enrich() function from the GALLO package. Similar to GO terms and KEGG pathways, QTLs were considered enriched when the FDR was < 0.05 and more than one QTL was described in the SheepQTLdb.

A flowchart summarizing the methodology applied in the current study is available in Supplementary Fig. 3.

## Supplementary Information

Supplementary Information.

## Data Availability

The whole-genome bisulfite sequencing data are available at European Nucleotide Archive (ENA) repository with accession number PRJEB56595 (https://www.ebi.ac.uk/ena/browser/view/PRJEB56595).
