# Supplementary material for: Intergenerational impact of dietary protein restriction in dairy ewes on epigenetic marks in the perirenal fat of their suckling lambs
Source: Sci Rep. 2023 Mar 16;13:4351. doi: 10.1038/s41598-023-31546-3 (PMC10020577; doi:10.1038/s41598-023-31546-3)
Supplement: Supplementary file 1 — Supplementary Information. [file 41598_2023_31546_MOESM1_ESM.zip › Supplementary_Table1.docx]

Supplementary Table 1: Descriptive statistics for the age (in days), half carcass weight (HCW) and half-carcass percentage of perirenal and cavitary fat for the 34 Assaf lambs born from the dams subjected to the nutritional protein restriction challenge (NPR) or the control (C) dams.

| Sample | Age (days) | HCW (kg) | Percentage of perirenal and cavitary fat | NutChal or Control group | FatGroup |
| --- | --- | --- | --- | --- | --- |
| Male_1 | 21 | 3.1 | 3.694 | C | High-PCF |
| Male_2 | 27 | 2.82 | 3.567 | C | High-PCF |
| Male_3 | 25 | 2.76 | 3.336 | NPR | High-PCF |
| Male_4 | 25 | 2.56 | 3.322 | NPR | High-PCF |
| Male_5 | 35 | 2.62 | 3.260 | NPR | High-PCF |
| Male_6 | 27 | 3.22 | 3.104 | NPR | High-PCF |
| Male_7 | 26 | 3.2 | 2.974 | NPR | - |
| Male_8 | 36 | 2.52 | 2.884 | C | High-PCF |
| Male_9 | 31 | 2.56 | 2.760 | C | High-PCF |
| Male_10 | 24 | 3.26 | 2.733 | NPR | - |
| Male_11 | 27 | 3.00 | 2.648 | NPR | - |
| Male_12 | 27 | 2.78 | 2.552 | NPR | - |
| Male_13 | 37 | 2.44 | 2.281 | NPR | - |
| Male_14 | 22 | 2.84 | 2.224 | C | - |
| Male_15 | 18 | 2.96 | 2.220 | NPR | - |
| Male_16 | 22 | 2.98 | 2.146 | C | - |
| Male_17 | 24 | 2.48 | 2.144 | C | - |
| Male_18 | 29 | 2.84 | 2.124 | NPR | - |
| Male_19 | 22 | 2.82 | 2.050 | C | - |
| Male_20 | 32 | 2.36 | 2.047 | NPR | - |
| Male_21 | 23 | 3 | 2.028 | C | - |
| Male_22 | 29 | 2.82 | 1.996 | C | - |
| Male_23 | 25 | 2.88 | 1.969 | NPR | Low-PCF |
| Male_24 | 19 | 3.8 | 1.936 | C | - |
| Male_25 | 19 | 3.12 | 1.930 | C | - |
| Male_26 | 20 | 2.76 | 1.856 | C | - |
| Male_27 | 22 | 3.34 | 1.797 | C | Low-PCF |
| Male_28 | 28 | 2.52 | 1.730 | NC | Low-PCF |
| Male_29 | 27 | 2.62 | 1.717 | C | Low-PCF |
| Male_30 | 22 | 2.66 | 1.684 | C | Low-PCF |
| Male_31 | 20 | 2.88 | 1.646 | NC | Low-PCF |
| Male_32 | 17 | 2.86 | 1.417 | C | Low-PCF |
| Male_33 | 16 | 3.16 | 1.365 | NC | Low-PCF |
| Male_34 | 21 | 2.9 | 1.152 | NC | - |

C: lambs which were born from control dams; NPR: lambs which were born from nutritional protein restriction dams; High-PCF: lambs which were assigned, within each NutChal group to the high-fat group; Low-PCF: lambs which were assigned, within each NutChal group to the low-fat group.
