# Supplementary material for: Intergenerational impact of dietary protein restriction in dairy ewes on epigenetic marks in the perirenal fat of their suckling lambs
Source: Sci Rep. 2023 Mar 16;13:4351. doi: 10.1038/s41598-023-31546-3 (PMC10020577; doi:10.1038/s41598-023-31546-3)
Supplement: Supplementary file 1 — Supplementary Information. [file 41598_2023_31546_MOESM1_ESM.zip › Supplementary_Table2.docx]

Supplementary Table 2: Mapping statistics for the whole genome bisulfite sequencing data from the perirenal fat of the Assaf suckling lambs included in the present study.

| Sample | Percentage of mapped reads (%) | Uniquely mapped reads | Unmapped reads | Total number of reads | %CG | %CHG | %CHH |
| --- | --- | --- | --- | --- | --- | --- | --- |
| NC_H_1 | 74.7932 | 199057570 | 67086202 | 266143772 | 71.87 | 1.45 | 1.562 |
| NC_H_2 | 69.193 | 127913124 | 56951086 | 184864210 | 70.823 | 1.399 | 1.462 |
| NC_H_3 | 68.622 | 152152263 | 69573092 | 221725355 | 71.797 | 1.372 | 1.426 |
| NC_H_4 | 69.2809 | 135776566 | 60203108 | 195979674 | 71.884 | 1.39 | 1.446 |
| NC_L_1 | 67.4524 | 124387601 | 60020435 | 184408036 | 71.373 | 1.395 | 1.453 |
| NC_L_2 | 65.5584 | 120860603 | 63494951 | 184355554 | 71.386 | 1.438 | 1.51 |
| NC_L_3 | 69.0879 | 128929056 | 57686930 | 186615986 | 70.495 | 1.436 | 1.508 |
| NC_L_4 | 68.61 | 143670867 | 65731404 | 209402271 | 71.224 | 1.366 | 1.415 |
| C_H_1 | 75.5818 | 153164517 | 49482902 | 202647419 | 71.731 | 1.44 | 1.544 |
| C_H_2 | 70.9802 | 130466775 | 53340477 | 183807252 | 71.149 | 1.426 | 1.497 |
| C_H_3 | 70.2453 | 130049921 | 55086893 | 185136814 | 71.703 | 1.41 | 1.47 |
| C_H_4 | 68.3268 | 125181640 | 58028450 | 183210090 | 70.746 | 1.397 | 1.465 |
| C_L_1 | 70.4151 | 130736150 | 54928693 | 185664843 | 71.628 | 1.409 | 1.484 |
| C_L_2 | 70.9833 | 131028068 | 53561890 | 184589958 | 71.686 | 1.389 | 1.452 |
| C_L_3 | 69.9271 | 129591609 | 55732313 | 185323922 | 71.491 | 1.39 | 1.449 |
| C_L_4 | 72.6195 | 134966698 | 50887893 | 185854591 | 71.762 | 1.467 | 1.523 |

NPR: Nutritional challenge; C: Control; H: High fat; L: Low fat
