# Supplementary material for: Intergenerational impact of dietary protein restriction in dairy ewes on epigenetic marks in the perirenal fat of their suckling lambs
Source: Sci Rep. 2023 Mar 16;13:4351. doi: 10.1038/s41598-023-31546-3 (PMC10020577; doi:10.1038/s41598-023-31546-3)
Supplement: Supplementary file 1 — Supplementary Information. [file 41598_2023_31546_MOESM1_ESM.zip › SupplementaryFigure1.docx]

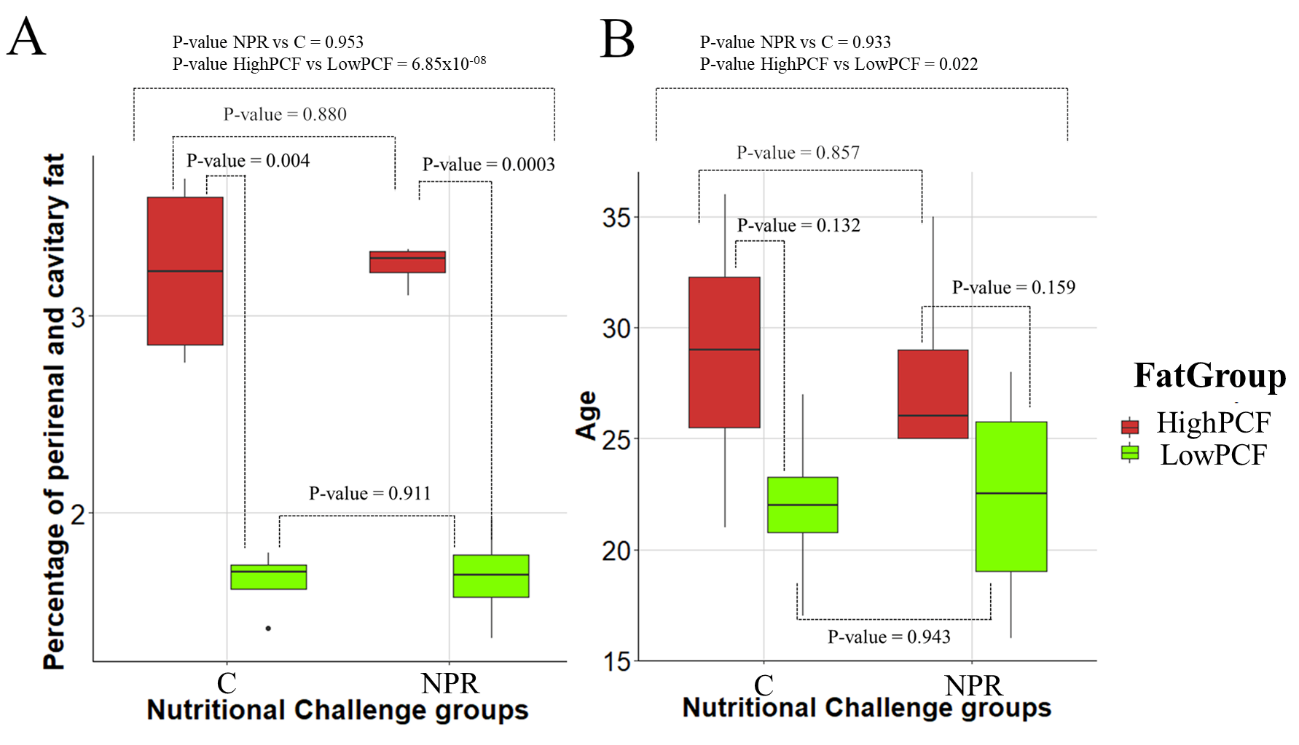


**Supplementary Figure 1:** Box-plot comparing the distribution of the percentage of perirenal and cavitary fat (A) and age in days (B) between nutritional protein restriction (NPR) and control (C) groups, high (HighPCF, in red) and low (LowPCF, in green) perirenal and cavitary fat groups, and between fat groups (HighPCF and LowPCF) within NPR and C groups. The dotted lines connect the comparison groups and show the p-values for the t-test comparing the means between groups.
