# Supplementary material for: Intergenerational impact of dietary protein restriction in dairy ewes on epigenetic marks in the perirenal fat of their suckling lambs
Source: Sci Rep. 2023 Mar 16;13:4351. doi: 10.1038/s41598-023-31546-3 (PMC10020577; doi:10.1038/s41598-023-31546-3)
Supplement: Supplementary file 1 — Supplementary Information. [file 41598_2023_31546_MOESM1_ESM.zip › SupplementaryFigure2.docx]

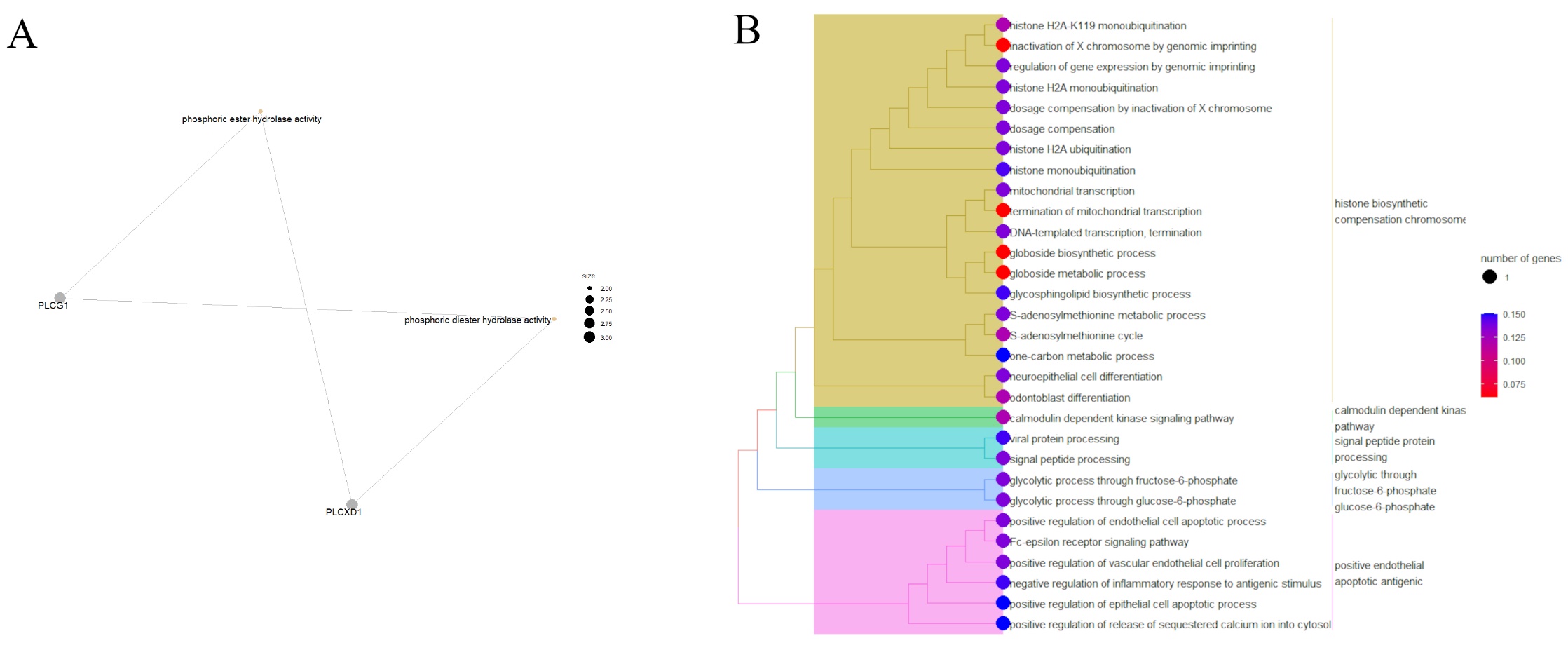


**Supplementary Figure 2:** Functional analysis for the genes harboring differentially methylated regions for the Nutchal term of the multifactorial model. A) Network composed of genes (gray circles) and gene ontology (GO) terms (yellow circles) showing the functional connection between the genes harboring DMRs identified for the NutChal term. B) Functional grouping tree diagram for the annotated GO terms. Each color in the dendrogram represents a functional group obtained after estimating the Jaccard correlation coefficient. The area of the circles represents the number of genes assigned to each GO term, and the color of the circle indicates the p-value estimated for each GO term.
