# Supplementary material for: Intergenerational impact of dietary protein restriction in dairy ewes on epigenetic marks in the perirenal fat of their suckling lambs
Source: Sci Rep. 2023 Mar 16;13:4351. doi: 10.1038/s41598-023-31546-3 (PMC10020577; doi:10.1038/s41598-023-31546-3)
Supplement: Supplementary file 1 — Supplementary Information. [file 41598_2023_31546_MOESM1_ESM.zip › SupplementaryFigure3.docx]

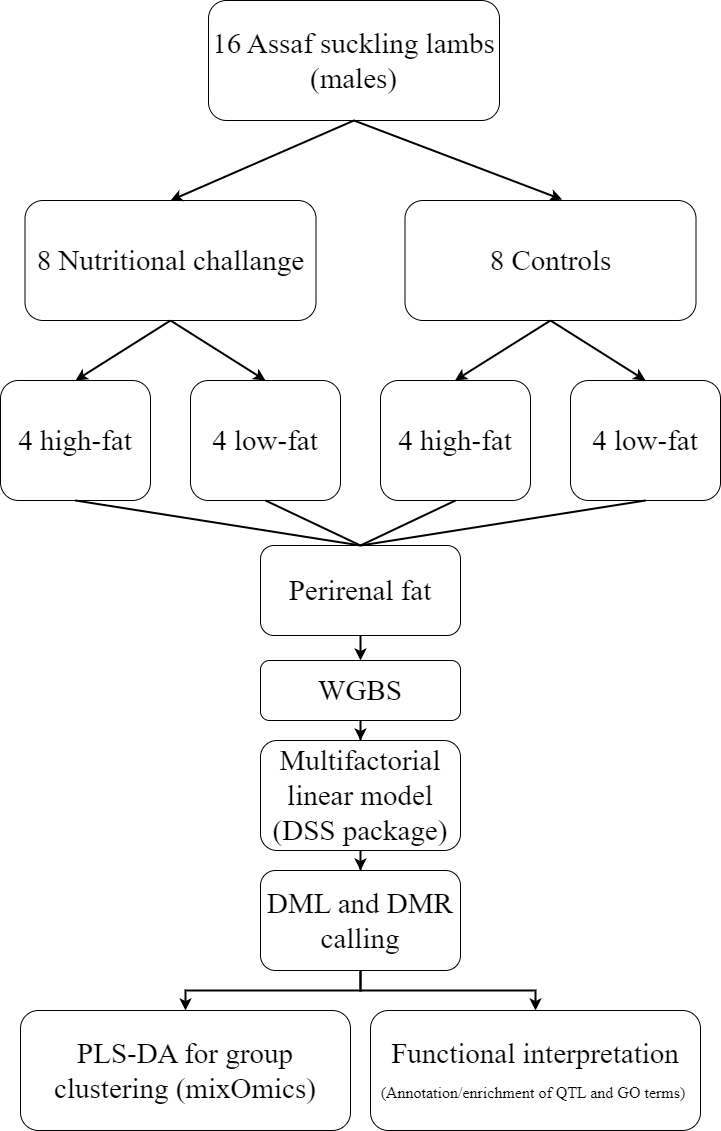


**Supplementary Figure 3:** Flowchart describing the general experimental design for the identification of differential methylated loci (DMLs) and regions (DMRs) in the perirenal fat of Assaf suckling lambs with high and low amounts of perirenal ad cavitary fat, which are the progeny of nutritional challenge or control ewes.
