# Supplementary material for: Intergenerational impact of dietary protein restriction in dairy ewes on epigenetic marks in the perirenal fat of their suckling lambs
Source: Sci Rep. 2023 Mar 16;13:4351. doi: 10.1038/s41598-023-31546-3 (PMC10020577; doi:10.1038/s41598-023-31546-3)
Supplement: Supplementary file 1 — Supplementary Information. [file 41598_2023_31546_MOESM1_ESM.zip › SupplementaryTable8.docx]

Supplementary Table 8. Ingredients and chemical composition of the diet provided to the two groups of ewes during the nutritional challenge: control group (C), and nutritional protein restriction group (NPR).

| Diet Composition | C | NPR |
| --- | --- | --- |
| Ingredients (g/kg) |  |  |
| Maize grain | 400 | 503 |
| Barley grain | 300 | 377 |
| Soybean meal 47 | 180 | ----- |
| Wheat bran | 60 | 60 |
| Lard | 10 | 10 |
| Molasses (beet) | 20 | 20 |
| Minerals and vitamins | 30 | 30 |
| Chemical composition  ) |  |  |
| Dry matter (DM; g/kg) | 869 | 870 |
| Ash (g/kg DM) | 61.0 | 51.8 |
| Crude protein (g/kg DM) | 182 | 105 |
| Neutral detergent fibre (g/kg DM) | 119 | 121 |
| Acid detergent fibre (g/kg DM) | 46.5 | 39.1 |
| Acid detergent lignin (g/kg DM) | 6.6 | 8.5 |
| Ether extract (g/kg DM) | 33.8 | 43.8 |
| Gross energy (kcal/kg DM) | 4396 | 4346 |
